# Sestrin2 Modulates AMPK Subunit Expression and Its Response to Ionizing Radiation in Breast Cancer Cells

**DOI:** 10.1371/journal.pone.0032035

**Published:** 2012-02-20

**Authors:** Toran Sanli, Katja Linher-Melville, Theodoros Tsakiridis, Gurmit Singh

**Affiliations:** 1 Research Department, Juravinski Cancer Center, Department of Pathology and Molecular Medicine, McMaster University, Hamilton, Ontario, Canada; 2 Department of Oncology, McMaster University, Hamilton, Ontario, Canada; Wayne State University School of Medicine, United States of America

## Abstract

**Background:**

The sestrin family of stress-responsive genes (SESN1-3) are suggested to be involved in regulation of metabolism and aging through modulation of the AMPK-mTOR pathway. AMP-activated protein kinase (AMPK) is an effector of the tumour suppressor LKB1, which regulates energy homeostasis, cell polarity, and the cell cycle. SESN1/2 can interact directly with AMPK in response to stress to maintain genomic integrity and suppress tumorigenesis. Ionizing radiation (IR), a widely used cancer therapy, is known to increase sestrin expression, and acutely activate AMPK. However, the regulation of AMPK expression by sestrins in response to IR has not been studied in depth.

**Methods and Findings:**

Through immunoprecipitation we observed that SESN2 directly interacted with the AMPKα1β1γ1 trimer and its upstream regulator LKB1 in MCF7 breast cancer cells. SESN2 overexpression was achieved using a Flag-tagged SESN2 expression vector or a stably-integrated tetracycline-inducible system, which also increased AMPKα1 and AMPKβ1 subunit phosphorylation, and co-localized with phosphorylated AMPKα-Thr127 in the cytoplasm. Furthermore, enhanced SESN2 expression increased protein levels of LKB1 and AMPKα1β1γ1, as well as mRNA levels of LKB1, AMPKα1, and AMPKβ1. Treatment of MCF7 cells with IR elevated AMPK expression and activity, but this effect was attenuated in the presence of SESN2 siRNA. In addition, elevated SESN2 inhibited IR-induced mTOR signalling and sensitized MCF7 cells to IR through an AMPK-dependent mechanism.

**Conclusions:**

Our results suggest that in breast cancer cells SESN2 is associated with AMPK, it is involved in regulation of basal and IR-induced expression and activation of this enzyme, and it mediates sensitization of cancer cells to IR.

## Introduction

In various malignancies including breast cancer, mitogen activated signals can become constitutively activated leading to increased metabolism and genotoxic stress [1]. There are various cellular compensatory mechanisms that respond genomic stress, including the tumour suppressor p53, which suppresses cell growth and propagation through the induction of numerous target genes [2]. Some products of p53 activation that are important in mediating stress-signalling include AMP-activated protein kinase (AMPK), Tuberous sclerosis 2 (TSC2), and sestrin1/2 (SESN1/2) [3], [4]

Sestrins (SESN) are a small family of stress-sensitive genes that are conserved across several species including *Caenorhabditis elegans, Drosophila melanogaster*, and mammals [5], [6]. Mammals express 3 different SESN family members characterized as SESN1-3. SESN1 and SESN2 were classified as members of the growth arrest and DNA damage (GADD) genes family that can regulate cell growth and viability under different cellular pressures [7], [8]. SESN3 was identified shortly after SESN2 through *in silico* analysis and was found to be a target of the forkhead transcription factors (FoxO) family [9], [10]. SESN also exhibit antioxidant properties and can inhibit intracellular ROS through restoration of overoxidized peroxiredoxins, the enzymes involved in sequestering H_2_0_2_ [5]. More recently, SESN have been shown to modulate important physiological signalling events that are independent of their redox function [11]. The *Drosophila* ortholog of sestrin (dSESN) is a negative feedback regulator of the target of rapamycin (TOR) through AMPK regulation, and dSESN deletion from flies leads to the accumulation of age-associated pathologies [6]. Conversely, mammalian SESN1/2 was shown to act as a scaffolding protein and form an active complex with AMPK and TSC2 to block mammalian-TOR (mTOR) signalling in response to genotoxic stress [4]. Furthermore, SESN2 also plays a role in the regulation of autophagy and exhibits tumour suppressive proprieties [12], [13].

AMPK is a heterotrimeric enzyme that is comprised of a catalytic α-subunit, as well as β and γ regulator subunits [14]. There are multiple isoforms of each AMPK subunit (α1, α2, β1, β2, γ1, γ2, and γ3) that allow for up to 12 different heterotrimeric AMPK combinations, each containing one of the α, β, and γ subunits [15]. However, the expression of these various AMPK subunits are tissue specific [15], [16]. For example, the catalytic AMPKα1 subunit is primarily found in endothelial cells, nerves, and smooth muscle [17]. Conversely, the other catalytic AMPKα2 subunit is mainly restricted to skeletal muscle and myocardial tissue [17]. AMPK acts as a fuel gauge by maintaining the ratio of cellular AMP/ATP. Metabolic stressors such as hypoxia, heat shock, and glucose deprivation can also activate AMPK [18], [19], as well as upstream kinases such as liver kinase B1 (LKB1). LKB1 is a tumour suppressor that is mutated in Peutz-Jeghers syndrome and can regulates AMPK by directly phosphorylating it on its Thr172 residue of the catalytic α subunit to increase AMPK activity [15], [20].

Radiation therapy is a common cancer treatment, and recently our laboratory has described that ionizing radiation (IR) can activate AMPK in various cancer cell lines [21]. Exposure to IR causes DNA damage, which in turn activates the kinase ataxia-telangiectasia mutated (ATM) to facilitate cell cycle arrest through stabilization of p53 [22]. IR has also been reported to enhanced expression of SESN1/2 [8] and modulate protein synthesis [23], all in an attempt to repair DNA damage if possible, or induce apoptosis. Here we present evidence that SESN2 not only activates AMPK, but also regulates the expression of AMPK subunits. In addition, we show that SESN2 mediates IR-induced AMPK expression and facilitates radiosensitization of breast cancer cells.

## Results

### SESN2 associates with AMPKα1β1γ1 and increases its phosphorylation in MCF7 cells

**Figure 1:**
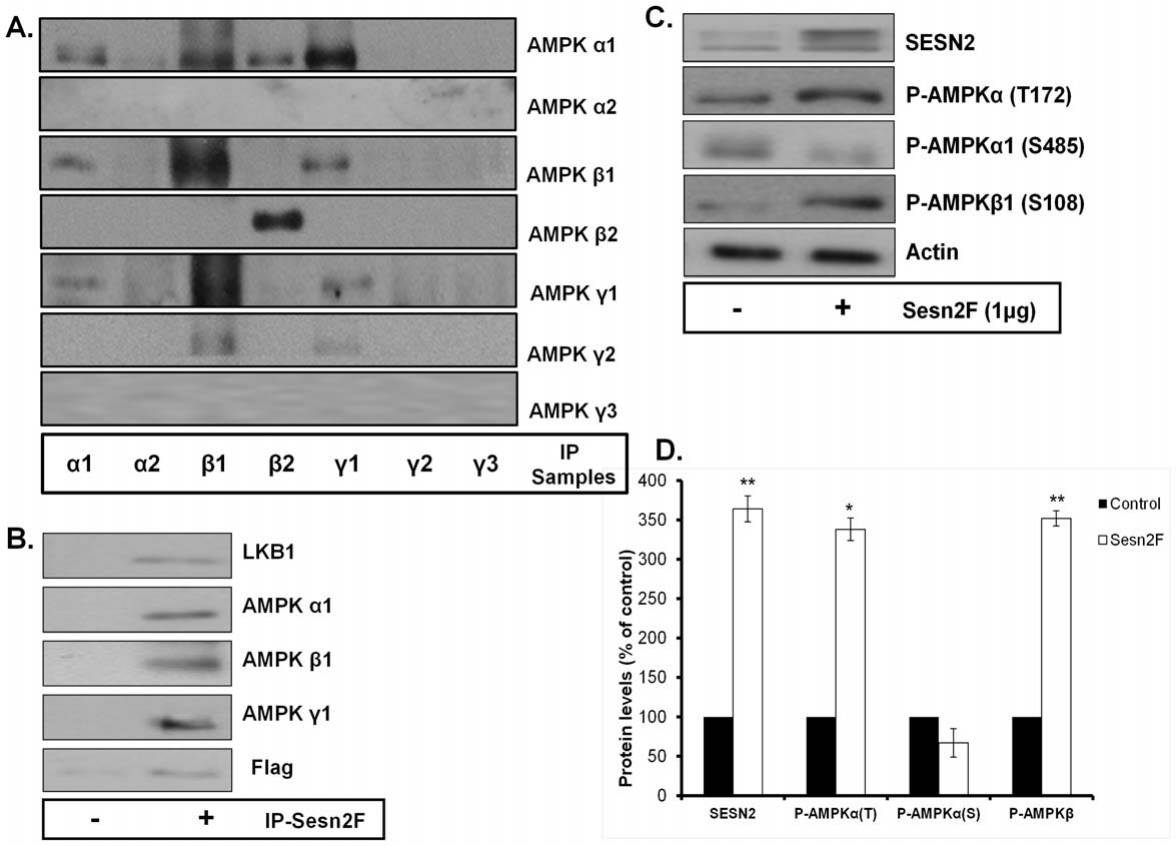
SESN2 interacts and regulates AMPKα1β1γ1 activity in MCF7 cells. (**A**) MCF7 cells were plated into a 10 cm dish and grown until fully confluent. The cells were then lysed with lysis buffer and immunoprecipitation was preformed with the indicated AMPK subunit antibodies. These samples were then subjected to western blotting with AMPK subunit antibodies. A representative immunoblot from 3 independent experiments is shown. (**B**) Cells were transfected with 1 µg empty Flag vector (−) or 1 µg Sesn2F (+). Forty eight hours later the cells were lysed and immunoprecipitation was preformed with an anti-Flag antibody. The samples were then subjected to western blotting with the indicated antibodies. (**C**) MCF7 cells were treated with 1 µg empty Flag vector (−) or 1 µg Sesn2F (+). Forty eight hours later the cells were lysed and subjected to western blotting with the indicated phosphorylated AMPK antibodies. (**D**) The results from (**C**) were quantitated and expressed as the mean and SE from 3 independent experiments (* = P<0.05 and ** = P<0.01 compared to control).

To examine the effect of SESN2 modulation on AMPK expression and activity, we first identified the most prominent AMPK heterotrimeric complex in MCF7 breast cancer cells by performing serial immunoprecipitations with antibodies against each AMPK subunit (α1–2, β1–2, and γ1–3), followed by immunoblotting (Figure 1A). AMPKα1, shown to be the major α-subunit in MCF7 cells [4] was highly associated with both AMPKβ1 and AMPKγ1 subunits. Conversely, the α2-subunit of AMPK was not detected by western blotting in these cells (Figure 1A). AMPKβ1 and AMPKβ2 are both expressed in this cell line, and the β1-subunit shares a stronger affinity with α1 and γ1 AMPK subunits (Figure 1A). Of the three γ-AMPK subunits, only the γ1 isoform of AMPK was detected in MCF7 cells and contributes to the active AMPK heterotrimeric complex (Figure 1A). Thus, the main AMPK active complex in MCF7 cells is the α1β1γ1 heterotrimer.

To assess the potential interaction of SESN2 with the AMPKα1β1γ1 complex, we immunoprecipitated SESN2 from MCF7 cells that were transiently transfected with 1 µg of a SESN2-Flag tagged expression vector (Sesn2F, Figure 1B). In agreement with previous studies, we found that SESN2 associates with AMPKα1 [4], as well as AMPKβ1 and AMPKγ1 subunits (Figure 1B). Since LKB1 is the major upstream kinase for AMPK, we examined whether LKB1 associates in a complex with SESN2 and AMPK. Indeed, LKB1 was present in immunoprecipitated Sesn2F treated MCF7 lysates (Figure 1B).

The activation state of AMPK was then evaluated with antibodies that detect phosphorylation of AMPK on α-Thr172, α1-Ser485, and β1-Ser108 residues, which are markers of AMPK activity (α-Thr172 and β1-Ser108) and inhibition (α1-Ser485) [24]. Transfection of MCF7 cells with 1 µg of Sesn2F led to a significant increase in AMPKα Thr172 and AMPKβ1 Ser108 phosphorylation levels and reduced AMPKα1 Ser485 phosphorylation, indicating an overall upregulation in the state of AMPK activation (Figure 1C–D).

### Subcellular distribution of SESN2 and activated AMPK

**Figure 2:**
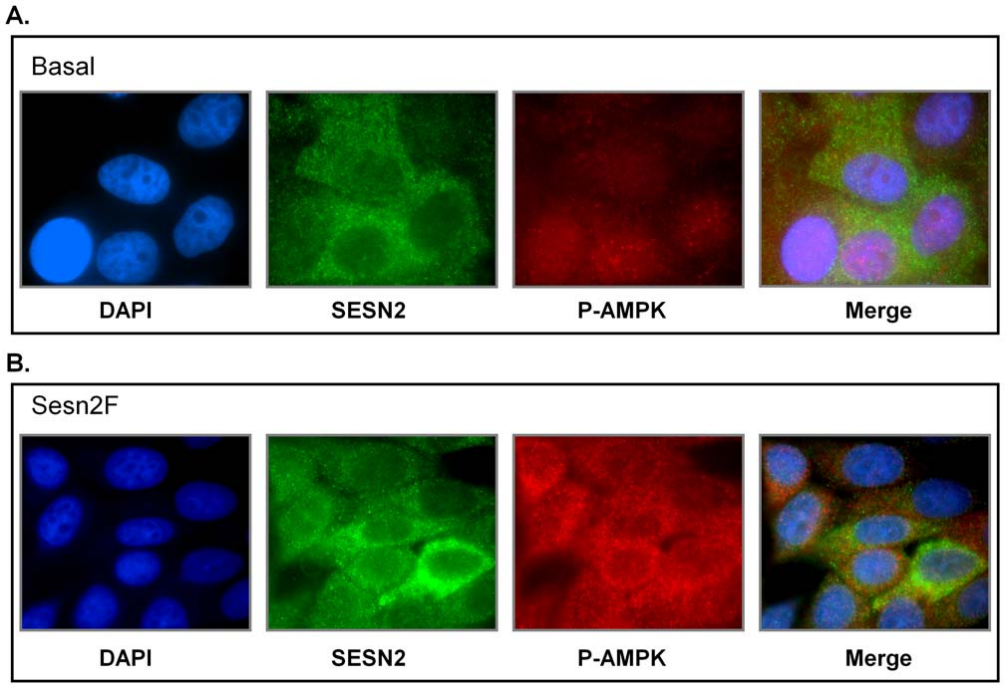
SESN2 is found in close proximity with active AMPK in the cytoplasm of MCF7 cells. (**A**) Untreated MCF7 cells were fixed and stained with DAPI (blue), SESN2 (green), or P-AMPKα (red). (**B**) MCF7 cells were transfected with 1 µg Sesn2F vector (Sesn2F) for 24 h, followed by fixation and staining with DAPI (blue), SESN2 (green), or P-AMPK (red). The cells were then imaged at 40× and merged images are displayed on the right. Representative images from 3 independent experiments are shown.

Although SESN2 has been described to interact with AMPK, the cellular localization of this interaction has not been identified. To address this we utilized immunoflourescence microscopy with antibodies against total SESN2 and phosphorylated Thr172-AMPKα (P-AMPK) in MCF7 cells (Figure 2A–B). Cells were transfected with either empty-Flag vehicle (control), or 1 µg Sesn2F for 48 h before fixation and labeling with the indicated antibodies. In control cells, SESN2 was detected mainly in the cytoplasm. On the other hand, phosphorylated AMPKα (P-AMPK) levels were low, showing very faint distributed in both nuclear and cytoplasmic cellular compartments (Figure 2A). However, in cells treated with Sesn2F both SESN2 and phosphorylated AMPK (P-AMPK) levels were enhanced, with SESN2 remaining largely in the cytoplasm. In addition, SESN2 overexpression led to a redistribution of active AMPK that shared close proximity with SESN2 in the cytoplasm (Figure 2B).

### SESN2 enhances AMPK subunit expression

To explore the effects of SESN2 on AMPK and LKB1 expression, we preformed a SESN2 dose-response (0.05–1 µg Sesn2F) transfection experiment in MCF7 cells to identify the optimal SESN2 levels required to modulate AMPK (Figure s1). A dose-dependent increase in SESN2 expression was achieved by increasing concentrations of Sesn2F cDNA (Figure s1A). However, the increase in AMPK subunit and LKB1 expression did not follow the same pattern, with noticeable effects on protein expression achieved at a dose as low as 0.05 µg Sesn2F. However, phosphorylation of AMPK and its downstream AMPK substrate, Acetyl CoA Carboxylase (P-ACC), a marker of AMPK activity, exhibited a dose-response enhancement with increasing concentrations of Sesn2F cDNA treatment that was parallel to that of SESN2 expression (Figure s1B).

To further validate these findings we utilized MCF7 Tet-OFF SESN2 cells that have conditional SESN2 overexpression via removal of doxycyclin (Dox) from their growth medium (Figure s1C–D). With the exception of AMPKγ1, we observed significant increases in SESN2, LKB1, AMPKα1, AMPKβ1, P-AMPKα (Thr172), and P-ACC levels following 24 h of Dox removal from the MCF7 Tet-OFF SESN2 media (Figure s1C–D). In contrast, SESN2 expression was associated with only a trend for increased AMPKγ1 levels that was not statistically significant (Figure s1C–D). In addition, we measured the mRNA levels of SESN2, LKB1, and AMPKα1β1γ1 following 24 h Dox withdrawal and observed a very significant increase in AMPKα1 levels (785±47% fold compared to control, P<0.01, Figure s1E). In addition, the mRNA levels of SESN2, LKB1, and AMPKβ1 were significantly enhanced (191±23%, 221±35%, and 171±22% fold compared to control respectively, P<0.05, Figure s1E).

### SESN2 plays a role in IR-induced AMPK activity/expression

**Figure 3:**
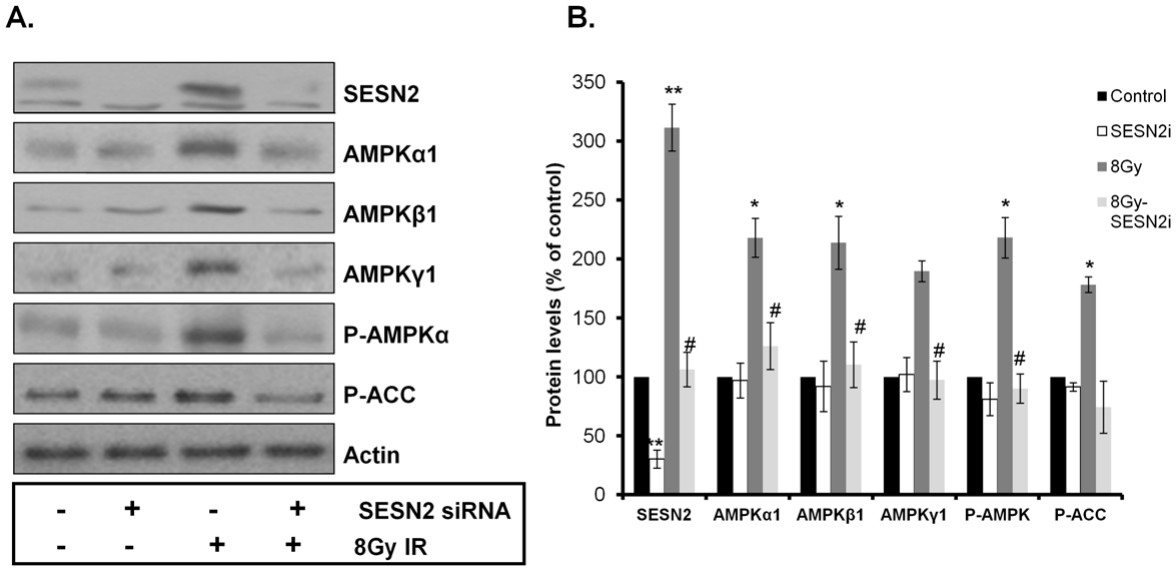
IR-induced expression and activity of AMPK is dependent on SESN2. (**A**) MCF7 cells were treated with SESN2 siRNA for 48 h prior to being exposed to a single dose of 8Gy IR. The cells were lysed 24 h after IR and western blotting was preformed with the indicated antibodies. A representative immunoblot from 3–4 independent experiments is shown. (**B**) The results from (**A**) were quantitated and expressed as the mean and SE from 4 independent experiments (* = P<0.05 compared to control, ** = P<0.01 compared to control, # = P<0.05 compared to 8Gy IR).

We hypothesized that radiation-induced AMPK activity/expression is dependent on SESN2. To examine this, we used siRNA against SESN2 in MCF7 cells that were treated with 8Gy of IR (Figure 3). SESN2 siRNA was added 48 h prior to a single dose of 8Gy IR. Twenty four hours after IR, the cells were lysed and the protein expression and phosphorylation of AMPK was evaluated (Figure 3A–B). SESN2 siRNA alone did not significantly affect basal protein levels of AMPK subunits. MCF7 cells exhibited a significant increase in SESN2 and AMPK subunit expression 24 h after IR (8Gy), as well as enhanced AMPKα and ACC phosphorylation (Figure 3A–B). However, the IR-induced increase in expression of all main AMPK subunits in MCF7 α1, ß1 and γ1 and AMPKα-T172 phosphorylation was attenuated in cells that were pre-treated with siRNA against SESN2 (Figure 3A–B), indicating that SESN2 plays a role in mediating IR-induced AMPK regulation.

### Enhanced SESN2 inhibits pro-survival pathways and sensitizes MCF7 cells to IR through AMPK

**Figure 4:**
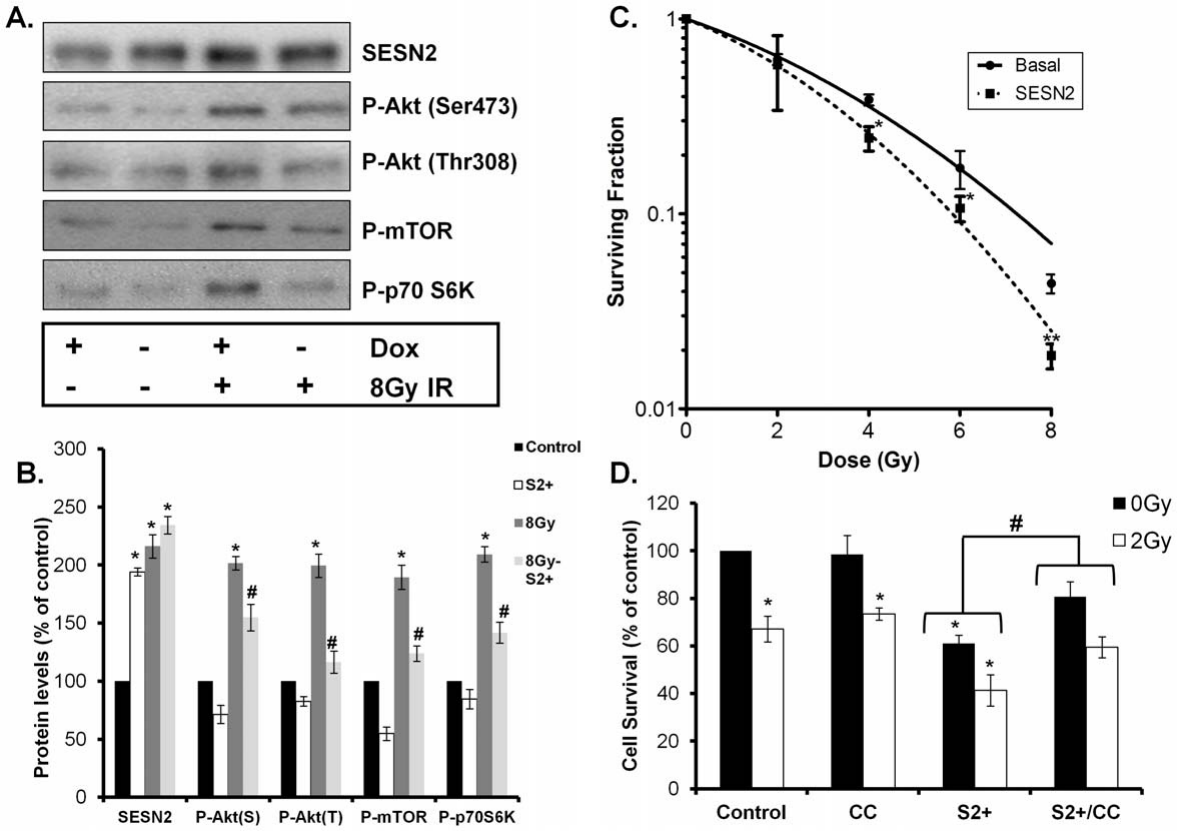
SESN2 in combination with IR modulates pathways of pro-survival and inhibits cell proliferation. (**A**) MCF7 Tet-OFF SESN2 cells were incubated in the presence (+) or absence (−) of Dox-containing medium for 24 h before exposure to 8Gy IR. Twenty four hours later the cells were lysed and subjected to western blotting with the indicated antibodies. A representative immunoblot from 3 independent experiments is shown. (**B**) The results from (**A**) were quantitated and expressed as the mean and SE from 3 independent experiments (* = P<0.05 compared to control and # = P<0.05 compared to 8Gy IR). (**C**) MCF7 Tet-OFF SESN2 cells were incubated in the presence (basal) or absence (SESN2) of Dox-containing medium for 24 h before exposure to the indicated doses of IR. Seven days later the cells were fixed and stained with mythelene blue and the clonogenic survival was calculated. Results from 3 independent experiments were averaged and presented as the mean and SE (* = P<0.05 and ** = P<0.01 compared to the corresponding IR treatment alone) and plotted on a logarithmic scale using the linear quadratic equation. The results were normalized so that both the untreated (basal) and SESN2 overexpressing cells (SESN2) start at the same point. (**D**) MCF7 Tet-OFF SESN2 cells were incubated in the presence (control) or absence (S2+) of Dox-containing medium and treated with or without 1 µM compound C (CC) for 24 h before exposure to 2Gy IR. Seven days later the cells were fixed and stained with mythelene blue and the clonogenic survival was calculated. Results from 3 independent experiments were averaged and presented as the mean and SE (* = P<0.05 compared to control and # = P<0.05 compared to S2+ alone).

We also examined the effect of SESN2 overexpression, IR, or the combined treatment on the activity of the Akt/mTOR survival pathway (Figure 4A–B). MCF7 tet-OFF SESN2 cells showed increased SENS2 levels after removal of doxycyclin and showed a trend for reduced phosphorylation of Akt, mTOR, and the mTOR substrate, p70-S6K (Figure 4A–B). Conversely, IR (8Gy) treatment led to a general stimulation of the Akt/mTOR pathway, but SESN2 overexpression 24 h prior to IR treatment significantly inhibited IR-induced activation of the Akt/mTOR signalling (Figure 4A–B).

To examine whether the effects of SESN2 overexpression and IR treatment, leading to inhibition of the Akt signaling pathway, influence cancer cell survival after IR, a clonogenic survival assay using radiation doses (0–8Gy) was conducted (Figure 4C). As expected, MCF7 cells that were treated with IR alone demonstrated a dose-dependent decrease in clonogenic survival. In addition, SESN2 overexpression had significant radiosensitizing effects particularly when combined with 4-8Gy IR (Figure 4C). Furthermore, to evaluate the role of AMPK in mediating SESN2-induced radiosensitization, we treated MCF7 cells with the AMPK chemical inhibitor compound C (CC) prior to SESN2 overexpression and exposure to 2Gy IR (Figure 4D). Enhanced SESN2 levels and 2Gy IR significantly inhibited MCF7 cell survival alone, and had an additive effect when both treatments were combined. On the other hand, CC did not significantly affect the survival of MCF7 cells alone, but showed a trend to reduce the ability of 2Gy IR to decrease breast cancer cell survival. Interestingly, CC significantly attenuated the ability of SESN2 overexpression to reduce cell survival alone and in response to IR.

## Discussion

SESN are a family of highly conserved, stress-inducible genes that can defend the cell against oxidative damage and oncogenic signalling [4], [25]. Recently, two members of this family, SESN1/2, have been found to play an important role in suppressing mTOR in response to genotoxic challenge through the regulation of AMPK signalling [4]. In addition, SESN2 has been implicated as a tumour suppressor that can inhibit angiogenesis and promote autophagy [2], underscoring the importance of elucidating the molecular mechanism by which SESN2 regulates pathways of metabolism and suvival. In this study, we have focused our efforts on investigating the relationship between SESN2 and its interaction with AMPK at the basal level, and in response to IR in breast cancer cells.

This study has identified that the primary active AMPK heterotrimeric complex in MCF7 cells is AMPKα1β1γ1. SESN2 was shown to form a protein complex with AMPKα1β1γ1 and LKB1 in MCF7 cells. In addition, we observed that SESN2 overexpression significantly enhanced the phosphorylation of AMPK on both α-Thr172 and β-Ser108 residues. SESN2 and LKB1 have been established to enhance α-Thr172 phosphorylation of AMPK [4], [26]. Conversely, β-Ser108 phosphorylation of AMPK is primarily achieved through auto-phosphorylation, and the ability of upstream kinases to directly target this site remains elusive [27]. Furthermore, SESN2 overexpression opposed AMPKα1-Ser485 phosphorylation, which has been identified as an inhibitory residue that blocks subsequent α-Thr172 phosphorylation by LKB1 [24]. Therefore, SESN2 may facilitate AMPK phosphorylation through a combination of recruitment of LKB1 and increased enzyme auto-phosphorylation.

Although the sub-cellular distribution of SESN2 may fluctuate between cytoplasmic and nuclear compartments, we observed that SESN2 is mainly localized in the cytoplasm of MCF7 cells. On the other hand, the localization of AMPK subunits varies dependent on the specific isoform, as well as their response to different stress stimuli [28], [29]. As we have observed in the past [21] and currently, there was a faint distribution of phosphorylated AMPK in both the nuclear and cytoplasmic compartments in unstimulated cells. However, SESN2 overexpression enhanced AMPK phosphorylation that was mainly prominent in the cytoplasm. Based on these observations it is likely that the majority of SESN2-AMPK interaction occurs within the cytoplasm of MCF7 cells.

Importantly, we also explored the effect of SESN2 overexpression on the total levels of LKB1, AMPK, P-AMPKα, and P-ACC. We observed for the first time that enhanced SESN2 expression alone can increase the mRNA and protein expression of the AMPK pathway (Figure s1). In particular, SESN2, P-AMPKα, and P-ACC exhibited a dose-dependent increase in expression following 0.05–1 µg Sesn2F treatment. However, the increase in LKB1 and AMPKα1β1γ1 expression in response to Sesn2F did not depict a classic dose-response pattern. These results suggest that LKB1/AMPK expression is very sensitive to changes in SESN2 levels (0–0.05 µg), while the activity of AMPK can be enhanced beyond a low concentration of Sesn2F (0.05–1 µg) (please refer to Figure s1A–B).

Moreover, utilization of MCF7 Tet-OFF SESN2 cells experienced similar increases in SESN2 as a low dose of Sesn2F, which also translated into enhanced LKB1/AMPK expression levels (Figure s1C–D). These cells also demonstrated significant increases in the mRNA levels of LKB1, AMPKα1, and AMPKβ1 suggesting that SESN2 may not only stabilize the association of these kinases, but also enhance their transcription as well (Figure s1E). The ability of SESN2 to alter gene transcription has not been investigated, but it has been speculated that SENS1/2 are part of a positive feedback loop that regulates p53 and AMPK expression and activity under times of genotoxic stress [4], [30]. For example, p53 is known to regulate SESN2 and AMPKβ1 gene expression [3], [8], while AMPKα is able to phosphorylate as well as transcriptionally regulate p53 in response to stress [31]. The most significant increase in mRNA levels with SENS2 overexpression in the AMPK pathway was AMPKα1, which supports the notion of AMPKα-mediated phosphorylation and stabilization of p53. In support of this notion, we have observed that Sesn2F treatment in MCF7 cells is also capable of increasing the phosphorylation and expression levels of p53 (Figure s2). Overall, there is a great deal of communication between the SESN2, p53, and the AMPK signalling pathway in response to stress stimuli that still requires investigation.

We have previously showed that 8Gy IR can acutely activate AMPK in multiple cancer cell lines [21]. However, the long-term effects of radiation or the influence of SESN on AMPK expression was not examined. In this study, we utilized IR as an agent to enhance SESN2 and found that not only was SESN2 levels increased 24 h-post 8Gy IR, but the expression and activity of the AMPK active complex (AMPKα1β1γ1) was elevated as well. Conversely, the IR-induced increase in AMPK and P-ACC expression was attenuated in MCF7 when they were pre-treated with siRNA against SESN2. We also observed the same trend in radiated A549 lung cancer cells, where IR-induced AMPK activity and expression was prevented with SESN2 siRNA (Figure s3). Taken together, these results suggest that IR induces a prolonged increase in SESN2 levels, which may be required for the sustained expression of the AMPK complex (α1β1γ1) in response to stress-stimuli.

Finally, we examined the effect of enhanced SESN2 on pathways of pro-survival that are affected by IR. Increased SESN2 expression inhibited Akt phosphorylation on both Ser473 and Thr308 residues in MCF7 Tet-OFF SESN2 cells, which are required for full Akt activation [32]. In addition, the phosphorylation of mTOR and its downstream substrate p70-S6K was also decreased with enhanced SESN2, validating the role of SESN2 as a negative regulator of mTOR signalling. IR (8Gy) treatment alone increased the expression of Akt/mTOR in MCF7 Tet-OFF SESN2 cells. However, this effect was attenuated when IR was combined with SESN2 overexpression, which translated into significant enhancement of IR-induced cell cytotoxicity when clonogenic survival was measured. In addition, we observed that the capability of SESN2 to augment cell death alone and in response to IR was dependent of AMPK activity, as compound C attenuated the SESN2-mediated reduction in MCF7 cell survival.

This model of SESN2 overexpression has been previously reported to modulate cell viability depending on the type of stress condition [8]. Enhanced SESN2 via the MCF7 Tet-OFF system sensitizes cell to DNA damaging treatments like UV radiation, and as we have shown, IR. However, overexpression of SESN2 was also established to protect cells from apoptosis induced by glucose deprivation [8]. Interestingly, AMPK is also required for prolonged cell survival upon glucose withdrawal [18] and irradiation during starvation [33], reinforcing the relationship between SESN2 and AMPK to modulate cell survival under different cellular pressures.

**Figure 5:**
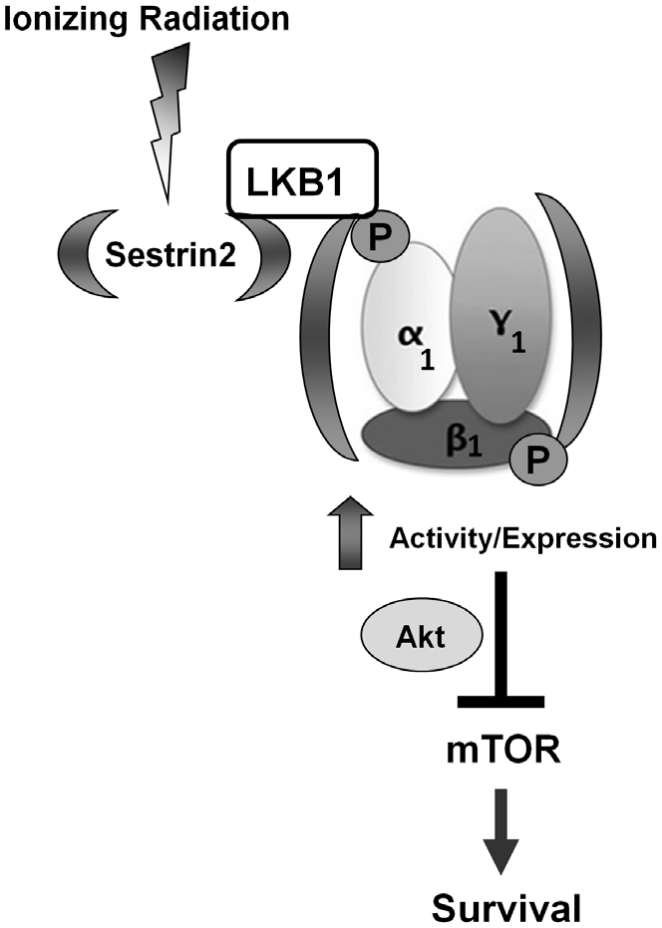
A proposed model of SESN2-mediated AMPK regulation in response to IR in MCF7 cells. In response to genotoxic stress (IR), SESN2 is enhanced and leads to the formation of an active LKB1/AMPKα1β1γ1 complex. SESN2 may stabilize the AMPK complex, or transcriptionally regulate AMPK and enhance its expression. Both SESN2 and AMPK may then coordinate mTOR suppression, which translates into enhanced IR-induced cancer cell killing.

Taken together, our model supports the notion that SESN2 is stress-activated gene that regulates AMPK activity by orchestrating recruitment of LKB1, as well as increasing LKB1/AMPKαβγ expression (Figure 5). In addition, we show for the first time that SESN2 blocks IR-induced Akt-mTOR signalling and acts as a radiation sensitizer in breast cancer cells (Figure 5). Future studies should elucidate the specific mechanism by which SESN2 phosphorylates AMPK, and examine its potential to act as a transcription factor that regulates metabolic gene expression.

## Materials and Methods

### Materials

DMEM media (5 mM glucose), RPMI media, fetal bovine serum (FBS), trypsin and antibiotic were purchased from Invitrogen (Burlington, ON, Canada). Antibodies against LKB1, phospho-AMPK α-subunit-(Thr172), phospho-AMPK α1-subunit-(Ser485), phospho-AMPK β-subunit-(Ser108), phospho-Acetly-CoA-Carboxylase (P-ACC), AMPKα1–2, AMPKβ1–2, AMPKγ1–3, phosphor-mTOR, phospho-p70-S6K, actin, and HRP-conjugated anti-rabbit secondary antibody were purchased from Cell Signalling (Mississauga, ON, Canada). Sestrin2 (SESN2) antibody was obtained from ProteinTech Group (Chicago, IL, USA). Polyvinylidene difluoride (PVDF) membrane was purchased from Pall Corporation (Port Washington, NY, USA). The FLAG-tag vector and antibodies were from Sigma (Toronto, ON, Canada). MCF7 cells were from the American Type Culture Collection (ATCC: Manassa, VA). The sestrin2 MCF7 tetracycline-OFF (Tet-OFF) cells was a kind gift from Dr. Michael Karin's laboratory (University of California, San Diego).

### Cell Culture and Treatments

MCF7 cells were grown in DMEM media that was supplemented with 10% (v/v) FBS and 1% (v/v) antibiotic-antimycotic. These cells were grown at 37°C as previously described [21]. Cells were treated with 2 to 8Gy IR using a clinical Linear Accelerator radiotherapy unit. The Tet-OFF MCF7 cells were maintained in standard DMEM growth medium supplemented with 0.5 µg/mL of doxycycline, and for SESN2 overexpression this medium was replaced with normal DMEM for 24 h [4]. Lipofectamin-2000 was used as a transfection reagent, and the cells were treated with plasmid vectors as previously described [4]. For siRNA transfection, cells were incubated with HiPerFect with or without siRNA against SESN2 for the indicated times, as per manufacturer's protocol [21].

### Clonogenic Assay

MCF7 Tet-OFF cells were subjected to clonogenic assays as described earlier [21]. In brief, 1000 cells were seeded into individual wells of a 6-well plate in triplicate 24 h before doxycycline withdrawal. Following 24 h of doxycycline removal, the cells were treated with a single dose of radiation (0–8Gy). After 7 days cells were fixed with mythelene blue and viable colonies (>50 cells) were counted. To assess radiation sensitization by SESN2, data was fitted to the linear quadratic equation using Graphpad Prism 5 software as previously described [34].

### Immunoprecipitation Assay

Following treatments, MCF7 cells were lysed in lysis buffer [20 mM Tris (pH 7.5), 150 mM NaCl, 1 mM EDTA, 1 mM EGTA, 1% Triton X-100, 2.5 mM Na_4_P_2_0_7_, 1 mM β-glycerolphosphate, 1 mM Na_3_VO_4_] containing one complete mini protease inhibitor cocktail tablet (Roche, Quebec Canada). 200 µL of lysate were then incubated with antibodies against the AMPK subunits or an anti-Flag antibody overnight, followed by the addition of 20 µL of protein A agarose beads (Sigma, Toronto, ON) for an additional 2 h. The samples were then repeatedly centrifuged and washed with lysis buffer prior to the addition of SDS-sample buffer and boiling.

### Immunoblotting

Twenty µg of protein was separated by SDS-PAGE and transferred to PVDF membranes as described earlier [21]. The primary antibody was detected with HRP-conjugated anti-rabbit or anti-mouse secondary antibody and ECL detection reagent.

### Real Time PCR

Total RNA was extracted from MCF7 cells, cDNA was prepared, and real time PCR was carried out as previously described [35]. The *AMPKα1* primer pairs were based on the PrimerBank (Harvard Medical School) ID 15214987a1. Primers for *SESN2* correspond to FOR: 5′-GCGAGATCAACAAGTTGCTGG-3′ and REV: 5′-ACAGCCAAACACGAAGGAGG-3′, and for *LKB1* FOR: 5′-GAGCTGATGTCGGTGGGTATG-3′, and REV: 5′-CACCTTGCCGTAAGAGCCT-3′, and for *AMPKβ1* FOR: 5′-GCATGGTGGCCATAAGACG-3′ and REV: 5′-GCGGGAGCTTTATCATTCAC-3′, and for *AMPKγ1* FOR: 5′-CATCCTCAAGAGACCCCAGA-3′ and REV: 5′-CACCGTTAGTCACCAAAGCA-3′. Primers used to amplify the *RPII* housekeeping gene were reported previously [35].

### Densitometry

The densitometry of immunoblots was performed using Image J software. Densitometry values are expressed as a percent change over the control value and are shown as mean ± SE of at least 3 independent experiments.

### Immunofluorescence Microscopy

Following treatments, cells were washed in PBS and fixed using 3% paraformaldehyde. The cells were labeled with the indicated primary antibodies and anti-mouse Alexa488 and anti-rabbit Alexa568 secondary antibodies were added the following day. The cells were then stained with DAPI and images were obtained as described previously [21].

### Statistical Analysis

Statistical analyses were performed using a student's T-test, or when appropriate, a one-way ANOVA with SPSS v16.0 software (Somers, NY). The results are presented as Mean ± SE of at least 3 separate experiments.

## Supporting Information

Figure S1 **SESN2 overexpression enhances AMPK and LKB1 levels.** (**A**) MCF7 cells were transiently transfected with 0.05–1 µg Sesn2F vector for 24 h, followed by cell lysis and western blotting with a SESN2 antibody (0 µg is defined as cells transfected with an empty-Flag vector). The results from western blotting were quantitated and expressed as the mean and SE from 3 independent experiments (* = P<0.05 and ** = P<0.01 compared to control). (**B**) MCF7 cells were transfected with 0.05–1 µg Sesn2F vector for 24 h, followed by cell lysis and western blotting with the indicated antibodies. (**C**) MCF7-tet-off cells were incubated in the presence (+) or absence (−) of Dox-containing medium for 24 h and subjected to western blotting with the indicated antibodies. (**D**) The results from (**C**) were quantitated and expressed as the mean and SE from 4 independent experiments (* = P<0.05 compared to control). (**E**) The SESN2, LKB1, and AMPK mRNA levels from MCF7-tet-off cells that were incubated in the presence (Dox +) or absence (Dox −) of Dox-containing medium for 24 h were measured. The results are presented as the mean and SE from 4 experiments (* = P<0.05 and ** = P<0.01 compared to control).
(TIF)

Figure S2 **SESN2 increases p53 phosphorylation and expression in MCF7 cells.** MCF7cells were treated with 1 µg Sesn2F for 48 h before lysis and western blotting with the indicated antibodies against p53. Actin was used as a loading control. A representative immunoblot from 3 independent experiments is shown.
(TIF)

Figure S3 **SESN2 is required for IR-induced AMPK activity and expression in A549 cells.** (A.) A549 cells were treated with SESN2 siRNA for 48 h before exposure to 8Gy IR. 24 h later the cells were lysed and subjected to western blotting with the indicated antibodies. (B.) The protein levels from (A.) were quantitated and expressed as the mean and SE of 3 independent experiments. ** = P<0.01 compared to control, * = P<0.05 compared to control, # = P<0.05 compared to 8Gy IR.
(TIF)
