# Supplementary figures and images for: Sestrin2 Modulates AMPK Subunit Expression and Its Response to Ionizing Radiation in Breast Cancer Cells
Source: PLoS One. 2012 Feb 20;7(2):e32035. doi: 10.1371/journal.pone.0032035 (PMC3282792; doi:10.1371/journal.pone.0032035)

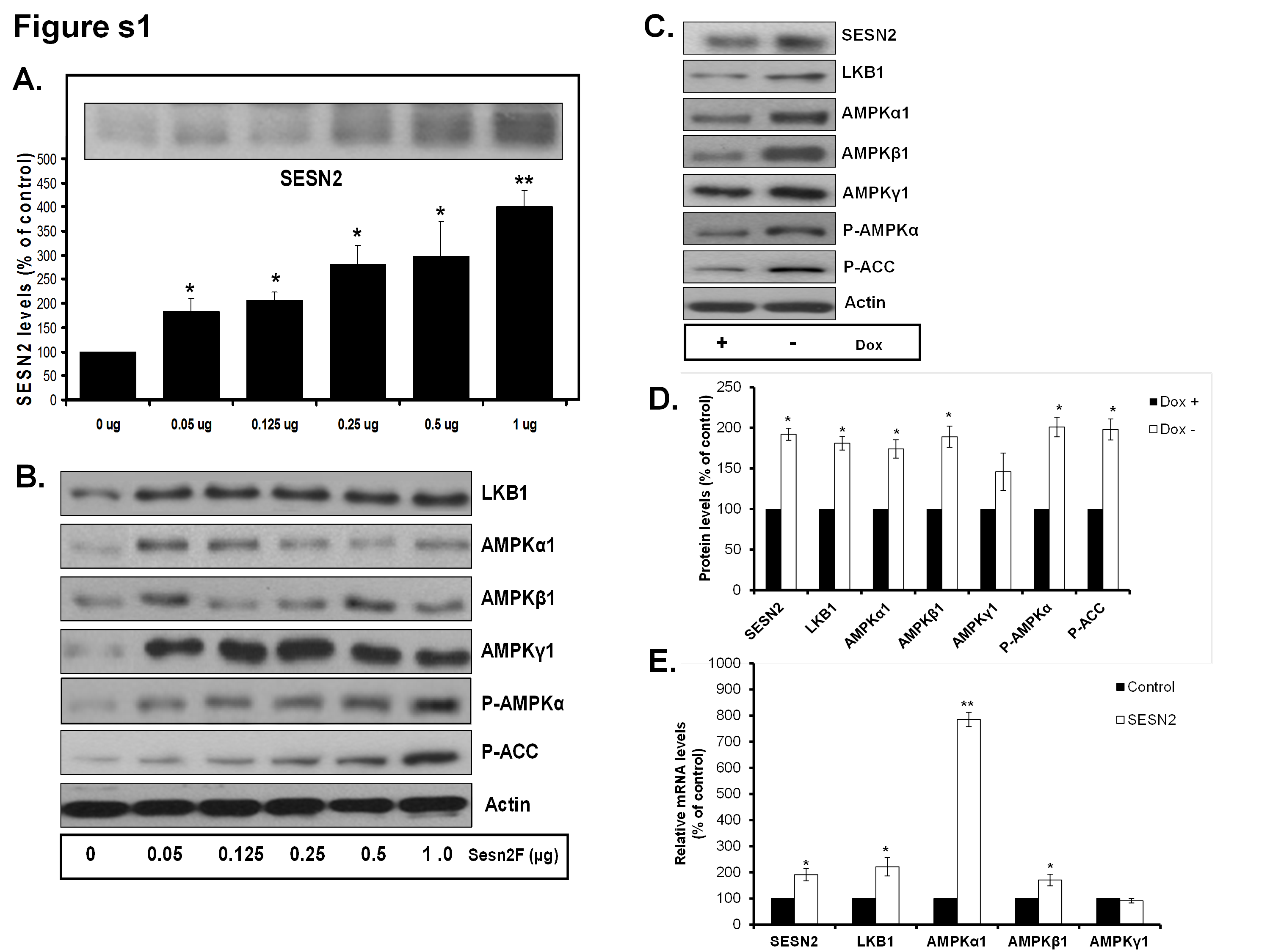

Supplement: Figure S1 — SESN2 overexpression enhances AMPK and LKB1 levels. (A) MCF7 cells were transiently transfected with 0.05–1 µg Sesn2F vector for 24 h, followed by cell lysis and western blotting with a SESN2 antibody (0 µg is defined as cells transfected with an empty-Flag vector). The results from western blotting were quantitated and expressed as the mean and SE from 3 independent experiments (* = P<0.05 and ** = P<0.01 compared to control). (B) MCF7 cells were transfected with 0.05–1 µg Sesn2F vector for 24 h, followed by cell lysis and western blotting with the indicated antibodies. (C) MCF7-tet-off cells were incubated in the presence (+) or absence (−) of Dox-containing medium for 24 h and subjected to western blotting with the indicated antibodies. (D) The results from (C) were quantitated and expressed as the mean and SE from 4 independent experiments (* = P<0.05 compared to control). (E) The SESN2, LKB1, and AMPK mRNA levels from MCF7-tet-off cells that were incubated in the presence (Dox +) or absence (Dox −) of Dox-containing medium for 24 h were measured. The results are presented as the mean and SE from 4 experiments (* = P<0.05 and ** = P<0.01 compared to control). (TIF) [file pone.0032035.s001.tif]

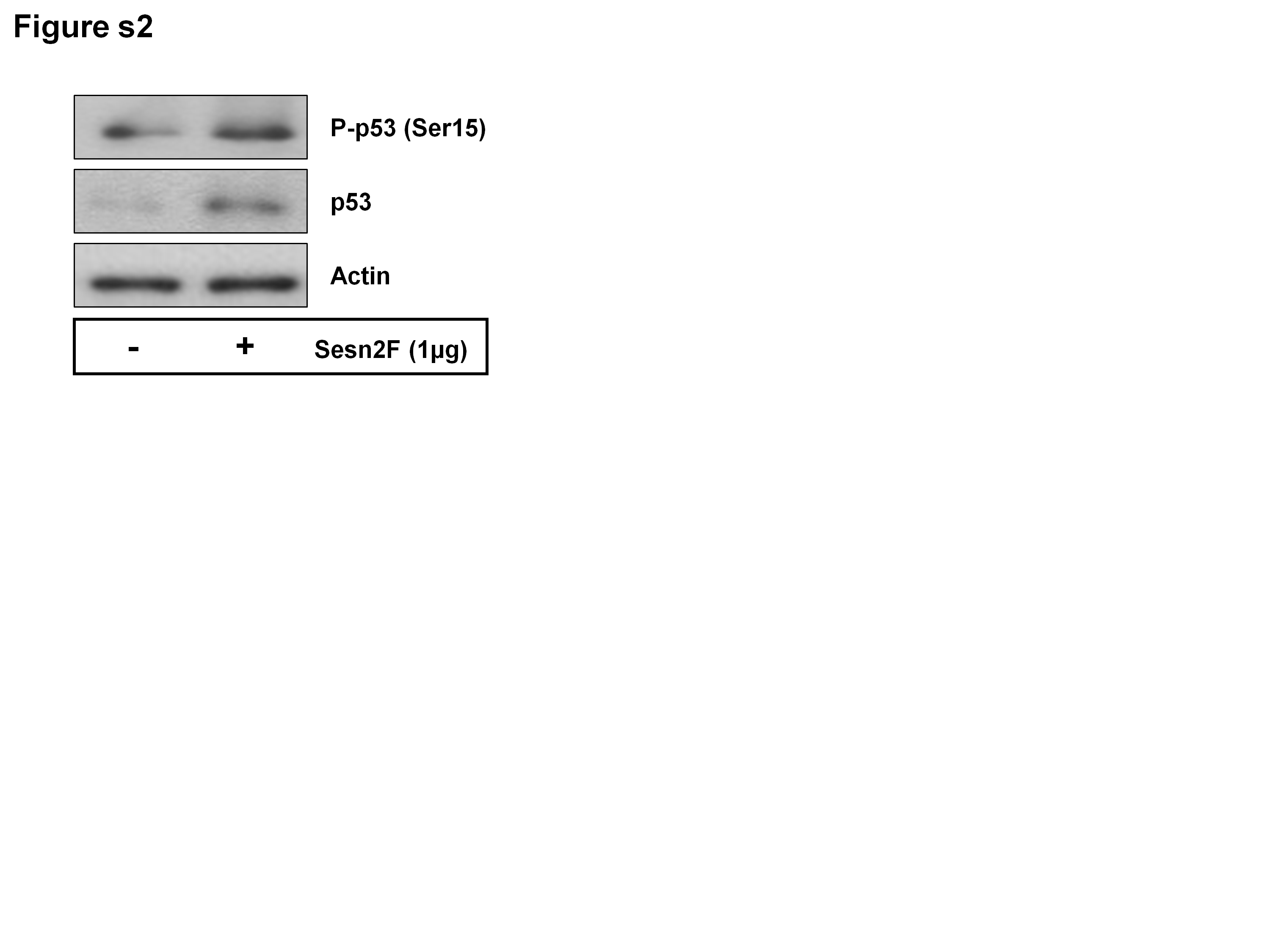

Supplement: Figure S2 — SESN2 increases p53 phosphorylation and expression in MCF7 cells. MCF7cells were treated with 1 µg Sesn2F for 48 h before lysis and western blotting with the indicated antibodies against p53. Actin was used as a loading control. A representative immunoblot from 3 independent experiments is shown. (TIF) [file pone.0032035.s002.tif]

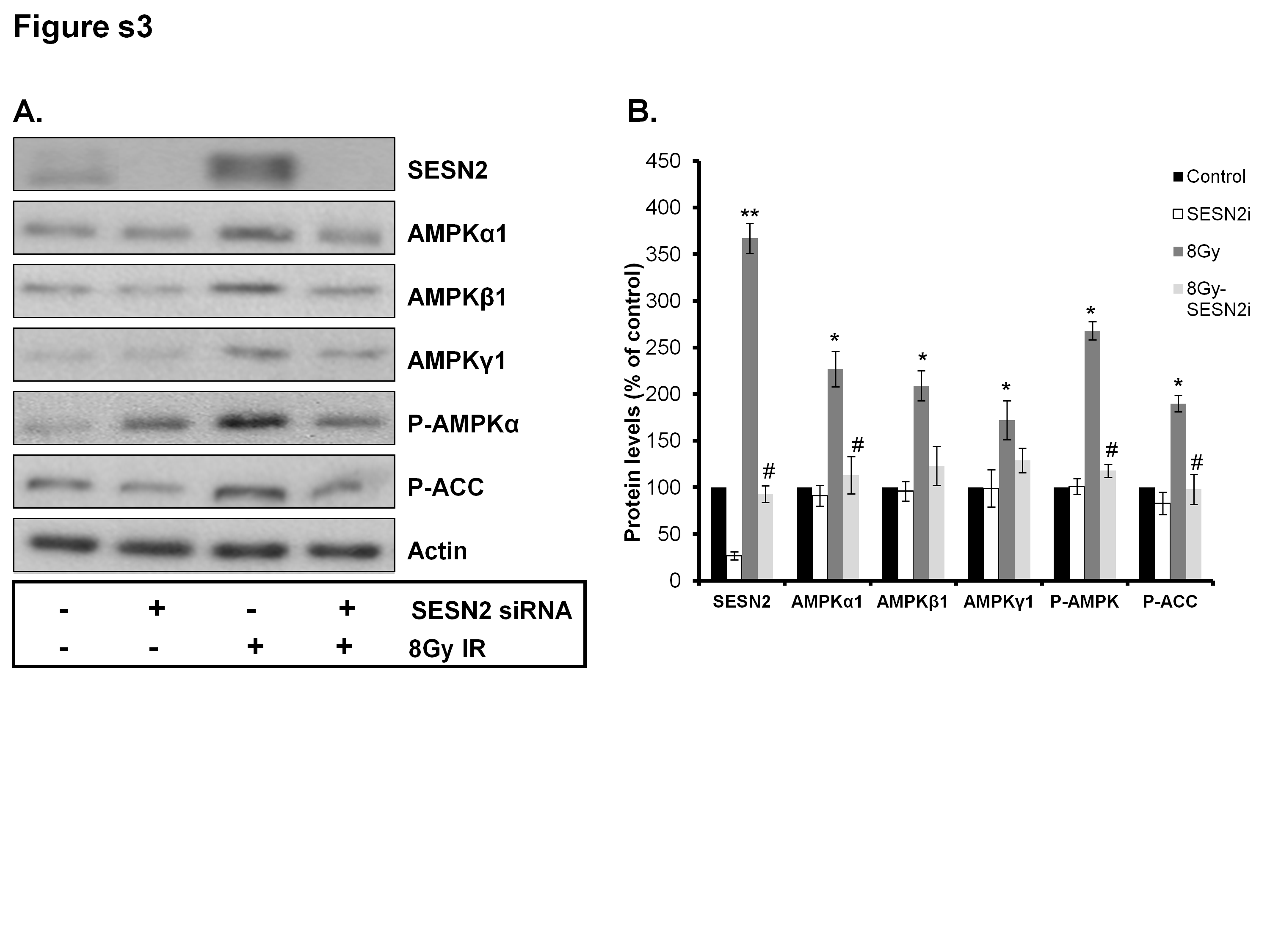

Supplement: Figure S3 — SESN2 is required for IR-induced AMPK activity and expression in A549 cells. (A.) A549 cells were treated with SESN2 siRNA for 48 h before exposure to 8Gy IR. 24 h later the cells were lysed and subjected to western blotting with the indicated antibodies. (B.) The protein levels from (A.) were quantitated and expressed as the mean and SE of 3 independent experiments. ** = P<0.01 compared to control, * = P<0.05 compared to control, # = P<0.05 compared to 8Gy IR. (TIF) [file pone.0032035.s003.tif]
